# A Study of the Effects of Daily Physical Activity on Memory and Attention Capacities in College Students

**DOI:** 10.1155/2018/2942930

**Published:** 2018-03-22

**Authors:** Dinh-Van Phan, Chien-Lung Chan, Ren-Hao Pan, Nan-Ping Yang, Hsiu-Chen Hsu, Hsien-Wei Ting, K. Robert Lai

**Affiliations:** ^1^Department of Information Management, Yuan Ze University, Taoyuan, Taiwan; ^2^Innovation Center for Big Data and Digital Convergence, Yuan Ze University, Taoyuan, Taiwan; ^3^University of Economics, The University of Danang, Danang, Vietnam; ^4^Department of Surgery & Orthopedics, Keelung Hospital, Ministry of Health and Welfare, Keelung, Taiwan; ^5^Faculty of Medicine, School of Medicine, National Yang-Ming University, Taipei, Taiwan; ^6^Department of Neurosurgery, Taipei Hospital, Ministry of Health and Welfare, New Taipei City, Taiwan; ^7^Department of Computer Science and Engineering, Yuan Ze University, Taoyuan, Taiwan

## Abstract

This study evaluated the relationship between daily physical activity (DPA) and memory capacity, as well as the association between daily activity and attention capacity, in college students in Taiwan. Participants (mean age = 20.79) wore wearable trackers for 106 days in order to collect DPA. These data were analyzed in association with their memory and attention capacities, as assessed using the spatial span test (SST) and the trail making test (TMT). The study showed significant negative correlations between memory capacity, time spent on the attention test (TSAT), calories burnt, and very active time duration (VATD) on the day before testing (*r* = −0.272, *r* = −0.176, *r* = 0.289, *r* = 0.254, resp.) and during the week prior to testing (*r* = −0.364, *r* = −0.395, *r* = 0.268, *r* = 0.241, resp.). The calories burnt and the VATD per day thresholds, which at best discriminated between normal-to-good and low attention capacity, were ≥2283 calories day^−1^, ≥20 minutes day^−1^ of very high activity (VHA) on the day before testing, or ≥13,640 calories week^−1^, ≥76 minutes week^−1^ of VHA during the week prior to testing. Findings indicated the short-term effects that VATD and calories burnt on the day before or during the week before testing significantly and negatively associated with memory and attention capacities of college students.

## 1. Introduction

Many studies have demonstrated that daily activity affects the physical and mental health of humans [1–6]. Therefore, from kindergarten to universities worldwide, physical education study or fitness events are held to improve student health and increase their learning efficiency. However, the search for strategies to improve health and increase study or work efficiency is laden with difficulties; there is a need to examine several, possibly interacting factors such as sex, age, environment, and living conditions, as well as examine historical data and investigate useful technologies.

Today, as quality of life has improved, people increasingly pay more attention to their personal health and personal development in order to enhance their life and improve their efficiency in terms of learning and work [7]. In conjunction, the development of mobile devices has also affected the lives of human beings. For example, special high-tech wearable equipment with sensors that can accurately collect information about human daily activity have increased in popularity and are now used widely. This has created an opportunity to study human activity more easily and accurately. The development of mobile technologies that utilize the Internet has brought people closer together; the world is narrowing, and almost everywhere is now in communication range via a mobile phone or a smart watch. Consequently, people have become more sedentary [8], due to objective and subjective reasons. In light of the above-mentioned trends and observations, we explored the relationship between daily activity and memory capability, as well as daily activity and attention capacity, in college students using wrist-worn trackers.

## 2. Methods

The study involved 39 participants who were first-year college students in Taiwan (15 females, mean age 20.79 years, SD 1.03), each of whom voluntarily signed an agreement to join the study. The participants wore wrist-worn trackers over a period of 106 days (from March 10, 2016, to June 23, 2016) to collect daily activity data under free-living conditions. The trackers collected data and synchronized to the Cloud via a Bluetooth 4.0 connection to a computer or mobile phone. Daily activity data of all participants were collected automatically from the Cloud and stored in an Impala data system, and our assessment tool was developed using Java programming language and an API (application programming interface). We performed SST and TMT, twice per participant, to examine participants' memory capacity and attention capacity; these test batteries were repeated 14 times during the study period. The results of the tests were divided into two levels: low values (<25th percentile) and normal-to-high values (≥25th percentile). This study was approved by the Institutional Review Board (IRB) with IRB number TH-IRB-0015-0016.

The wrist-worn tracker used in this study was the Fitbit Charge HR™ device manufactured by Fitbit Inc. This device can track daily personal activity and measures variables such as heart rate, calories burnt, steps taken, distance travelled, floors climbed, physical active minutes, exercise, and sleep characteristics. It has PurePulse LED lights that reflect onto the skin to detect capillary expansion based on blood volume change in order to measure heart rate [9, 10]. Activities were divided into the following levels according to the physical activity level (PAL): sedentary activity (1.0 ≤ PAL < 1.4), light activity (1.4 ≤ PAL < 1.6), fair activity (1.6 ≤ PAL < 1.9), and high activity (1.9 ≤ PAL < 2.5) [11]. Moderate-to-vigorous physical activity (MVPA) consisted of fair and high activities. The rate of calories burnt at rest just to maintain vital body functions such as breathing, heartbeat, and brain activity was calculated as the basal metabolic rate. The daily physical data collected were adjusted by gender, age, height, and weight [11, 12]. Therefore, we did not need to adjust data on an individual basis in this study.

**Figure 1:**
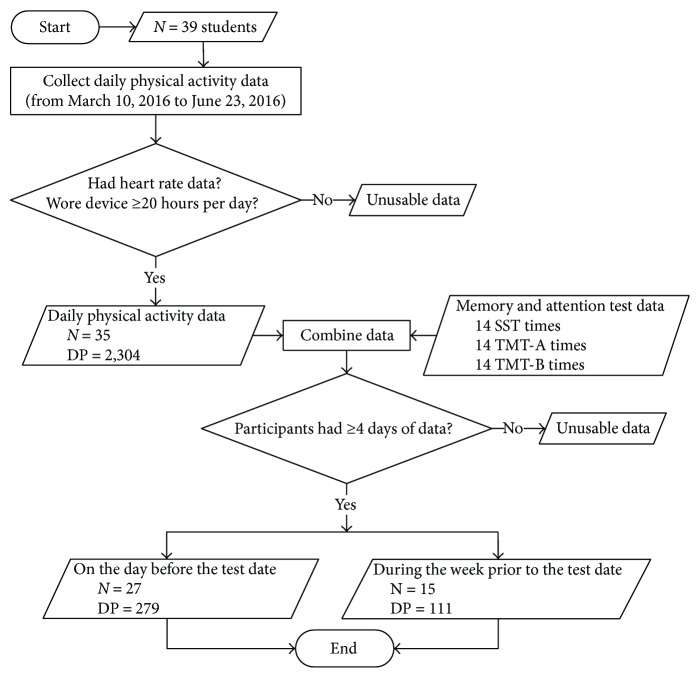
Study sample flowchart. DP: day-participant; SST: spatial span test; TMT: trail making test.

The attention capacity of the participants was evaluated using the TMT, which is one of the most popular neuropsychological tests. It can measure executive functions, such as visual search speed, scanning, mental flexibility, and speed of processing [13–16]. TMTs have been developed for use with computers and tablets, which have the advantages of simplicity, accuracy, and random transformation of positional repeatability measurement [17, 18]. A computerized version of TMT was also built for this study using Java programming language to automatically collect results with high accuracy time (Figure 1).

The TMT consists of two parts. TMT-A requires participants to click sequentially connecting 25 consecutive digits from 1 to 25 (1, 2,…, 25) displayed at random positions on the interface. TMT-B requires testers to click sequentially connecting alternate values between 13 numbers and 12 alphabet letters (A, B,…, L) (e.g., 1, A, 2, B, 3, and C), which are also displayed at random positions on the interface. The test result was recorded as the time required to complete the test [13], this time spent on the attention test (TSAT) representing attention capacity; that is, the longer the time taken, the lower the attention capacity [19].

The SST was a computerized version provided by Cambridge Brain Sciences (http://www.cambridgebrainsciences.com). It consists of squares that light up on the interface, which are used to assess memory capacity. A participant sees squares lighting up and clicks the sequence in the same order. If the participants respond incorrectly, the previous level is presented. The test is terminated when the participant responds incorrectly three consecutive times. The memory capacity of the participant was calculated as the maximum number of squares to which the participant responded correctly [20, 21].

According to the recommendations of the manufacturer, the trackers used in this study should be recharged after several days and taken off while bathing or swimming, so the raw data need filtering before analysis. This study was based on heart rate data collected every five seconds by the trackers, and the filter conditions were as follows: (1) data were excluded if heart rate data at the same time were lacking; (2) if data had not been collected for at least 20 hours per day, the participant was excluded—fourteen memory and attention test data were continuously combined with daily activity data on the day before the test date and data collected during the week prior to the test date; (3) after combining the data, participants for whom fewer than four days of data were obtained were excluded. Finally, this study included two data groups for analysis: data obtained on the day before the test date (*N* = 27; 279 DPs) and data obtained during the week prior to the test date (*N* = 15; 125 DPs). This study focuses only on short-term physical activities associated with attention and memory capacity of college students. We tried to determine which activity precisely on one day before the test date will immediately associate with memory and attention capacity. Therefore, we analyzed the physical activities one day before the test date. Nevertheless, there could be a chance that participants altered their behaviors in the day immediately before the test date. Hence, we also analyzed physical activities during one week prior to the test date to eliminate this factor.

### 2.1. Statistical Analysis

The memory and attention test data (fourteen measurements—once per week) were combined with the DPA data obtained on the day before the test date and during the week prior to the test date; so the final data were measured repeatedly, but the number of measurements for each participant was different. Therefore, the PROC MIXED model (SAS 9.4 program) was applied in this study to analyze the correlations of DPA with memory and attention capacities [22–26]. This model is recommended for repeated measures and missing data [27]. The mixed model used the maximum likelihood (ML), DDFM = KR, and the TYPE = UN specifies a general variance-covariance matrix. The correlation coefficient was estimated by VCORR, and the bootstrap method was used to estimate 95% confidence intervals (CIs). This study also employed IBM SPSS Statistics Version 22 program to analyze quartile and descriptive statistics, in addition to the linear trend test. The receiver operating characteristic (ROC) was used to calculate the optimal cut-off points for calories burnt and the VATD based on optimizing the difference between sensitivity and specificity.

## 3. Results and Discussion

There were 33 participants in our study initially; however, during the period of the study, six more participants joined, and one participant dropped out. After the data were filtered, there remained 35 participants (89.74%) who wore a tracker for greater than or equal to 20 hours per day, which therefore accounted for 2304 day-participants (DPs). These data continuously combined fourteen memory and attention tests on the day before the test date, and every participant had data for more than or equal to four days. The final data consisted of 27 (16 male, 69.23%) participants (279 DPs). The daily activity data were combined with data obtained from fourteen memory and attention tests during the week before the test date (who had at least seven days' data), and every participant had more than or equal to four days of data. The results represented 15 (10 male, 38.46%) participants in total (111 DPs).  Table 1 shows the means (SDs) of DPA, memory capacity, and attention capacity on the day before the test date and during the week prior to the test date.

A mixed-model analysis showed a significant negative correlation between memory capacity and calories burnt on the day before the test date and during the week prior to the test date [*r* = −0.272 (95% CI: −0.342, −0.160), *r* = −0.364 (95% CI: −0.476, −0.179), resp.]. The analysis results also showed a significant negative correlation between memory capacity and the VATD on the day before the test date and during the week before the test date [*r* = −0.176 (95% CI: −0.270, −0.079), *r* = −0.395 (95% CI: −0.524, −0.237), resp.] (Table 2).

Regarding attention capacity, the analysis results showed a significant positive correlation between the TSAT and calories burnt (meaning that there existed a negative correlation between attention capacity and calories burnt) on the day before the test date and during the week prior to the test date [*r* = 0.289 (95% CI: 0.207, 0.366), *r* = 0.268 (95% CI: 0.095, 0.361), resp.]. The study results also demonstrated a significant positive correlation between the TSAT and the VATD (meaning that there was a negative correlation between the VATD and attention capacity) on the day before the test date and during the week prior to the test date [*r* = 0.254 (95% CI: 0.164, 0.351), *r* = 0.241 (95% CI: 0.091, 0.405), resp.]. Additionally, MVPA on the day before the test date positively associated with the TSAT (and negatively associated with attention capacity), with *r* = 0.198 (95% CI: 0.090, 0.305) (Table 2).

**Figure 2:**
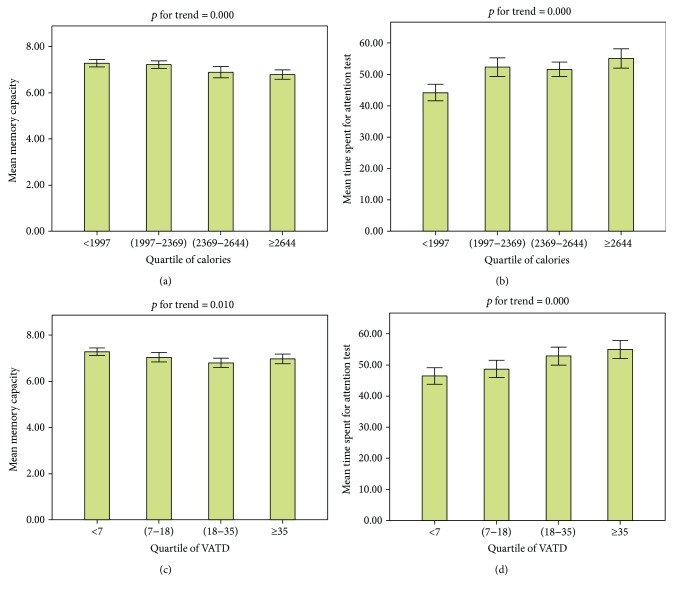
Mean of memory capacity and TSAT according to quartile of calories and the VATD on the day before the test date. (a) Memory capacity and calories, (b) TSAT and calories, (c) memory capacity and VATD, and (d) TSAT and VATD.

**Figure 3:**
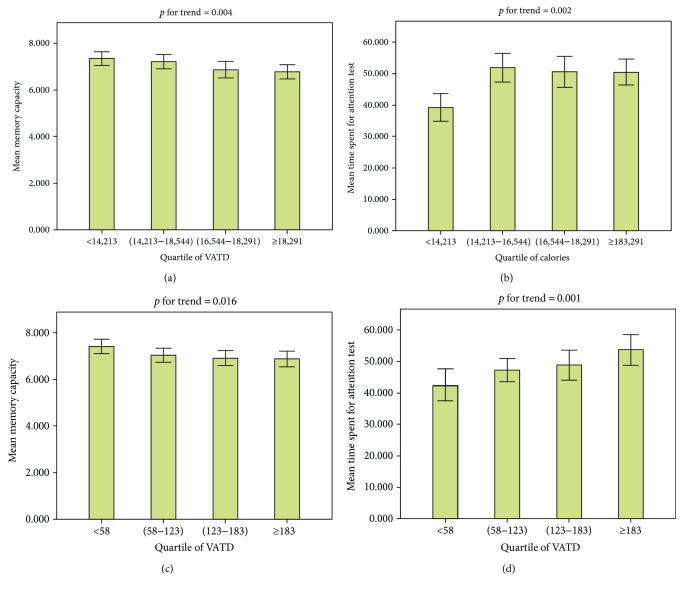
Mean of memory capacity and TSAT according to quartile of calories and VATD during the week prior to the test date. (a) Memory capacity and calories, (b) TSAT and calories, (c) memory capacity and VATD, and (d) TSAT and VATD.

Quartile analysis of physical activity also confirmed that a linear association existed between memory capacity and the TSAT and calories burnt, the VATD on the day before the test date (*p* for trend < 0.05) (Figure 2). A linear association also existed between memory capacity and the TSAT and calories burnt, the VATD during the week before the test date (*p* for trend < 0.05) (Figure 3).

Previous studies have indicated that daily moderate-to-vigorous physical activity (MVPA) positively affects the memory [28–30] and attention capacities of humans [31–34]. For example, higher academic performance is strongly and consistently related to a greater sedentary duration [35, 36]. Physical activity during the school day improves attention to tasks among elementary students [37, 38]. However, our study indicated that vigorous activity negatively associated with memory and attention capacities [39].

Galioto et al. studied 122 adults from the Longitudinal Assessment of Bariatric Surgery-2 parent project and identified weak correlations of self-reported aerobic physical activity with lower attention capacity (*r* = −0.21, *p* = 0.04) and execution capacity (*r* = −0.27. *p* < 0.01), and both self-reported aerobic physical activity and objectively determined MVPA min/week were negatively correlated with memory capacity (*r* = −0.20, *p* = 0.04; *r* = −0.46; *p* = 0.02, resp.) [40]. Indeed, our study of college students also showed that both the VATD on the day before the test date and the VATD during the week prior to the test date were negatively correlated with memory capacity [*r* = −0.176 (95% CI: −0.270, −0.079), *r* = −0.395 (95% CI: −0.524, −0.237), resp.], and both the VATD on the day before the test date and the VATD during the week prior to the test date were positively correlated with the TSAT (and negatively correlated with attention capacity) [*r* = 0.254 (95% CI: 0.164, 0.351), *r* = 0.241 (95% CI: 0.091, 0.405), resp.].

A study of 74 children (mean age = 8.6 years, SD = 0.58, 46% girls) from 7 schools in East Central Illinois, US, from October 2013 to 2014 indicated no significant associations between MVPA and inhibition, working memory, or academic achievement [41]. Another study of 80 typically developing children (aged 8–12 years, 44 girls) in The Netherlands also demonstrated no significant associations between MVPA and visual memory span or TMT [42]. A study of the Healthy Lifestyle in Europe by Nutrition in Adolescence from 2006 to 2007 indicated that adolescents' attention capacity test performances were significantly and positively associated with a longer time spent performing moderate activity or MVPA under free-living conditions (*p* < 0.05). Promoting MVPA may have a beneficial effect on attention capacity. That study used the d2 test of attention to assess attention capacity prior to the participants (273 adolescents, aged 12.5–17.5 years) being monitored in terms of daily activity under free-living conditions using GT1M devices for 7 days, 8 hours per day [43]. However, our study assessed 39 students (mean age 20.79 years, SD = 1.03, 38.46% female) over a period of 106 days and filtered subjects for whom data were available ≥20 hours per day under free-living conditions for analysis; the participants were tested 14 times during this period, and every participant included in the final analysis had more than or equal to four days' worth of data. Our results showed that students' memory and attention capacities were significantly and negatively associated with a longer VATD and higher calories burnt. We also share the same view as Vanhelst et al. [43] in that increasing the VATD of students may lead to fatigue, and hence reduced cognitive functions such as memory and attention capacities. Therefore, moderate daily activity or light activity may improve memory and attention capacities [36, 37].

Vanhelst indicated that spending more than 58 minutes per day in MVPA was associated with a better attention capacity [43]. However, in the short term, our study found that more than 2283 calories burnt and more than 20 minutes of VATD on the day before the test, and 13,640 calories burnt and 76 minutes of VATD during the week prior to the test date, were associated with a poorer attention capacity (Table 3). This VATD cut-off point was consequently recommended by the American Heart Association, in that adults should spend at least 75 minutes per week participating in vigorous physical exercise [44]. The reported association between lower attention capacities in the short-term following vigorous activity might suggest that it was due to fatigue [43]. A study of Davis and Bailey also indicated that fatigue during prolonged exercise obviously is influenced by central nervous system because prolonged exercise released ammonia into the blood that could alter central nervous system function [45]. Therefore, during the fatigue time, subjects may be less attentive and have less cognitive resources available to the individual concerned, and it requires additional time for recovery.

In this study, data were collected via wearable trackers in free-living conditions, and the advantage was continuous data collection without intrusion of subjects' daily lives. However, these data can be influenced by individual characteristics and external factors. For instance, physical activity level depends on demographic characteristics [46, 47]. Each participant may have different personal behavior and habit; therefore, personal physical activities are also different. The health of participants before the test such as sleepiness and napping also may affect their memory and attention capacities [48, 49] on which we had not tested before the test. In addition, the wearable trackers needed recharging after several days and taking off while participants were bathing and swimming.

It is likely that participants might have inadvertently altered their behavior while wearing the devices (as compared to the baseline when no such devices were being worn). These factors may influence the physical activity. However, this study indicated a convenient application based on wearable devices to monitor daily physical activities for getting memory and attention benefits.

## 4. Conclusions

Our exploratory study assessed the relationships between daily activity and memory capacity, and daily activity and the TSAT. Higher calories burnt or a greater VATD on the day before and during the week before a certain day is associated with a lower memory capacity and a lower attention capacity on that day. In addition, it was also found in this study that the calories burnt and the VATD on the day before (≥2283 calories, ≥20 minutes, resp.) and the calories burnt and the VATD during the week before (≥13,640 calories, ≥76 minutes, resp.) a certain day were associated with a poorer attention capacity.

Our results were obtained from an exploratory study and not a random control trial, and hence, no comparisons were made between two independent groups. As the study was performed in a free-living environment, it was influenced by external factors and personal physiological characteristics of the participants. The findings have little supporting evidence, and few studies have been performed that produced the same results. Further studies will be designed as random control trials in order to compare two groups and control external impacting factors in a free-living environment.

## Figures and Tables

**Table 1:** Descriptive statistics of DPA, memory capacity, and attention capacity.

	Measure	On the day before the test date	During the week before the test date
		*N* = 27 (Male = 16) DPs = 279 Mean (SD)	*N* = 15 (Male = 10) DPs = 111 Mean (SD)
Daily physical activity			
Calories	Calories	2347.27 (490.97)	16,435.97 (2920.77)
Steps	Steps	9084.68 (3467.87)	63,828.14 (21,894.72)
Distance	km	06.36 (02.45)	44.47 (15.54)
Floors	Floors	21.74 (13.96)	136.54 (68.24)
Elevation	Miles	65.74 (42.52)	412.97 (207.80)
Sedentary time duration	Minutes	795.42 (186.21)	5336.23 (759.71)
LATD	Minutes	168.80 (87.41)	1337.76 (411.19)
FATD	Minutes	22.25 (20.05)	176.52 (137.58)
VATD	Minutes	24.08 (22.75)	138.18 (109.42)
Memory and attention tests			
SST	Capability	07.04 (00.84)	07.05 (00.83)
TMT-A	Minutes	45.00 (10.58)	43.18 (11.03)
TMT-B	Minutes	56.59 (16.25)	53.13 (16.55)

DPs: day-participants; LATD: lightly active time duration; FATD: fairly active time duration; VATD: very active time duration; SST: spatial span test; TMT: trail making test.

**Table 2:** Correlation coefficients between DPA and the SST and TSAT.

Daily physical activity	On the day before the test date *N* = 279, *r* (95% CI)^∗^				During the week before the test date *N* = 111, *r* (95% CI)^∗^			
	SST	TMT-A	TMT-B	Mean of TMT	SST	TMT-A	TMT-B	Mean of TMT
Calories	−0.272 (−0.342, −0.160)	0.226 (0.136, 0.310)	0.288 (0.205, 0.358)	0.289 (0.207, 0.366)	−0.364 (−0.476, −0.179)	0.220 (0.038, 0.300)	0.270 (0.095, 0.373)	0.268 (0.095, 0.361)
Steps	−0.025 (−0.134, 0.078)	0.032 (−0.092, 0.155)	0.068 (−0.035, 0.170)	0.059 (−0.054, 0.168)	−0.041 (−0.226, 0.141)	−0.131 (−0.309, 0.041)	0.011 (−0.179, 0.210)	−0.050 (−0.239, 0.150)
Distance	−0.031 (−0.136, 0.077)	0.015 (−0.104, 0.137)	0.045 (−0.059, 0.151)	0.037 (−0.075, 0.149)	−0.098 (−0.252, 0.085)	−0.159 (−0.317, −0.011)	−0.040 (−0.205, 0.149)	−0.096 (−0.269, 0.077)
Floors	0.005 (−0.116, 0.113)	−0.082 (−0.186, 0.008)	−0.024 (−0.100, 0.056)	−0.051 (−0.130, 0.026)	0.111 (−0.033, 0.274)	−0.190 (−0.341, −0.053)	−0.049 (−0.189, 0.103)	−0.115 (−0.259, 0.028)
Elevation	0.005 (−0.117, 0.113)	−0.082 (−0.186, 0.009)	−0.024 (−0.100, 0.056)	−0.051 (−0.130, 0.026)	0.111 (−0.033, 0.274)	−0.190 (−0.341, −0.053)	−0.049 (−0.189, 0.102)	−0.115 (−0.259, 0.028)
Sedentary time duration	0.046 (−0.061, 0.150)	−0.038 (−0.137, 0.058)	−0.042 (−0.129, 0.043)	−0.044 (−0.135, 0.040)	−0.071 (−0.241, 0.094)	−0.078 (−0.222, 0.098)	−0.100 (−0.282, 0.051)	−0.098 (−0.265, 0.061)
LATD	−0.077 (−0.167, 0.019)	−0.073 (−0.161, 0.027)	−0.070 (−0.147, 0.014)	−0.077 (−0.154, 0.010)	−0.152 (−0.313, 0.041)	−0.208 (−0.369, −0.035)	−0.151 (−0.314, 0.052)	−0.190 (−0.359, 0.004)
FATD	0.062 (−0.059, 0.180)	0.059 (−0.059, 0.172)	0.051 (−0.079, 0.183)	0.059 (−0.068, 0.184)	0.092 (−0.044, 0.245)	−0.036 (−0.220, 0.142)	0.043 (−0.181, 0.298)	0.012 (−0.207, 0.252)
VATD	−0.176 (−0.270, −0.079)	0.222 (0.124, 0.327)	0.237 (0.146, 0.332)	0.254 (0.164, 0.351)	−0.395 (−0.524, −0.237)	0.188 (0.062, 0.332)	0.244 (0.081, 0.415)	0.241 (0.091, 0.405)
MVPA	−0.079 (−0.188, 0.031)	0.178 (0.069, 0.286)	0.183 (0.075, 0.290)	0.198 (0.090, 0.305)	−0.161 (−0.321, 0.020)	0.080 (−0.066, 0.231)	0.166 (−0.024, 0.381)	0.144 (−0.038, 0.343)

^∗^95% CI: 95% bootstrap confident interval; SST: spatial span test; TMT: trail making test; Mean of TMT: mean of TMT-A and TMT-B; LATD: lightly active time duration; FATD: fairly active time duration; VATD: very active time duration; MVPA: moderate-to-vigorous physical activity.

**Table 3:** Physical activity cut-off points to predict good attention capacity according to the ROC.

	On the day before the test					During the week before the test				
	Unit/d	SE	SP	AUC	*p* value	Unit/d	SE	SP	AUC	*p* value
Calories burnt (calories)	2283	0.649	0.676	0.698	0.000	13,640	0.917	0.593	0.794	0.000
VATD (minutes)	20	0.540	0.765	0.662	0.000	76	0.798	0.667	0.791	0.000

Unit/d: unit per day; SE: sensitivity; SP: specificity; AUC: area under the receiver operating characteristic curve.
